# We should be using nonlinear indices when relating heart-rate dynamics to cognition and mood

**DOI:** 10.1038/srep16619

**Published:** 2015-11-13

**Authors:** Hayley Young, David Benton

**Affiliations:** 1 Department of Psychology, University of Swansea, Wales, United Kingdom

## Abstract

Both heart rate (HR) and brain functioning involve the integrated output of a multitude of regulatory mechanisms, that are not quantified adequately by linear approximations such as means and standard deviations. It was therefore considered whether non-linear measures of HR complexity are more strongly associated with cognition and mood. Whilst resting, the inter-beat (R-R) time series of twenty-one males and twenty-four females were measured for five minutes. The data were summarised using time, frequency and nonlinear complexity measures. Attention, memory, reaction times, mood and cortisol levels were assessed. Nonlinear HR indices captured additional information, enabling a greater percentage of the variance in behaviour to be explained. On occasions non-linear indices were related to aspects for behaviour, for example focused attention and cortisol production, when time or frequency indices were not. These effects were sexually dimorphic with HR complexity being more strongly associated with the behaviour of females. It was concluded that nonlinear rather than linear methods of summarizing the HR times series offers a novel way of relating brain functioning and behaviour. It should be considered whether non-linear measures of HR complexity can be used as a biomarker of the integrated functioning of the brain.

The heart receives the brain’s commands through the central autonomic network^1^, with the prefrontal cortex playing a leading role^2^. Research has found that individual differences in heart rate variability (HRV) predict cognitive performance^3^, especially in tasks associated with the prefrontal cortex^4^. However, this research has focused on traditional methods of analysing interbeat (R-R) time series based on linearity and variance; such approaches are unable to detect subtle non-linear changes in interbeat intervals^5^. The present study demonstrated that linear and nonlinear HR indices are independently associated with cognition and mood, suggesting that nonlinear HR analysis can capture information not obtained using traditional linear methods. For the first time the relationship between HR nonlinearity and behaviour was shown to be sexually dimorphic, with nonlinear HR relating to behaviour in females but not males, presumably reflecting gender-associated differences in brain structure and functioning. It is suggested that the nonlinearity of the R-R interval is a marker of the brains ability to subtly and appropriately respond, both cognitively and emotionally, to minor change in environmental demands.

Although many physiological processes are known to be nonlinear, linear approximations are commonly used to describe them^6^. A good example is describing the R-R time series in terms of the standard deviation of the inter-beat interval. This gives rise to a basic measure of variability yet provides no information regarding the pattern or regularity of changes in the inter-beat intervals. For instance, the time series 5, 10, 5, 10 has the same variability as the time series 5, 5, 10, 10 but a different underlying pattern. Nonlinear analysis allows the quantification of this extra information. In fact, although the autonomic nervous system (ANS) activity has a substantial influence on the R-R interval^7^, other factors are important including thermoregulation^8^, endocrine factors^9^, adenosine^10^ circadian rhythm^11^ and level of fitness^12^. Simply, a classical linear HRV analysis is unable to reflect the many factors involved in the regulation of the heart.

In 1996, standards for the quantification of HRV were suggested^13^ which included indices from time and frequency domains. Since then, new methods based on systems and information theory and nonlinear dynamics have been proposed that quantify the complexity of the R-R time series. These include quantifying the fractal like structure of a time series^14^, entropy based measures^15^ and recurrence quantification analysis^16^. Several of these nonlinear indices have been related to disease states: for example, cardiac disease^17^, autonomic dysfunction in depression^18^ and other psychiatric diseases^19^. However, in healthy individuals the relationship between nonlinear indices of HRV and cognitive functioning has been largely ignored.

Another question which has been ignored is the possibility that there are sex differences in the control of heart-rate. Female and male brains differ both functionally and structurally^20^. One example is that compared to males, females have a higher percentage of grey matter even after normalising for brain size^21^. Interestingly, MRI based studies have shown that females have volumetrically larger orbitofrontal cortices than males^22^, an area of the brain thought to be involved in HRV^4^. Indeed, many studies have reported sex-related differences in HRV^23^ including those using nonlinear measures^24^. However, it is not known whether sex differences moderate the relationship between HRV and cognition. Studies to date have either: used entirely male samples^3^; have not considered the influence of sex^25^; or, have matched samples on gender in order to control for any differences^26^.

Therefore, the present study had two aims. Firstly, it sought to determine the relative association of linear and nonlinear HR indices to cognition and mood. Secondly, to see whether the sex of the subject moderated any relationships found. To address these questions forty nine (twenty-four males and twenty-five females) healthy participants were recruited. R-R interval measurements were recorded while participants rested quietly for five minutes listening to relaxing music. After the relaxation period, heart rate was no longer recorded and participants completed standardised questionnaires about their recent mood and stress levels. Participants then took a series of neuropsychological tests designed to assess cognitive domains known to be associated with HRV^4^ (namely working memory, focused attention and reaction times). Salivary cortisol measurements were also retrieved at baseline (upon arrival at the lab), after relaxation, and again after completion of the cognitive tests. The procedure is illustrated in Fig. 1. In line with previous findings^3^,^4^, it was hypothesised that linear HRV would be associated with measures of attention and mood, in particular ratings of depression. Further, it was predicted that adding nonlinear HR indices would increase the amount of variance explained.

## Statistical Analysis

Statistical analysis proceeded in three stages. In stage 1 HR indices were calculated which were then entered into a principal components analysis (stage 2). Finally, in stage 3 a series of hierarchical regressions were conducted, that determined the relative associations between the HR components (derived in stage 2) and aspects of cognition and mood.

### Stage 1 - Calculation of HR indices

R-R interval data were analysed using Kubios HRV Analysis Software 2.0 (The Biomedical Signal and Medical Imaging Analysis Group, Department of Applied Physics, University of Kuopio, Finland). Data were visually inspected for artefacts caused by ectopic beats, poor conductivity etc. A very low correction threshold was chosen for artefact correction (0.45 from local average) so not to distort natural variability. Less than 1% of beats were identified as artefacts, however, prior to the final analysis, two cases were removed based on very poor recording (both male) and two were removed based on abnormal HR responses (one male, one female). The latter cases were considered extreme outliers that without removal would have significantly altered the final analysis. These exclusions resulted in a remaining sample of twenty one males and twenty four females.

#### Time domain analysis

Time domain HRV indices included mean interbeat interval (R-R) (a measure of basic heart rate) and the standard deviation of normal to normal R-R interval (SDNN) (measures total variability in the series).

#### Frequency domain analysis

Spectral analysis was conducted to transform the time series into the frequency domain. The R-R interval series was converted to equidistantly sampled series by cubic spline interpolation at a rate of 4Hz. Welsh’s periodogram, which divides the R-R series into overlapping windows, was used to decrease the leakage effect, and the spectrum estimate was obtained by averaging the Fast Fourier Transform (FFT) spectra of these windowed segments. Average spectral power was estimated within the Low frequency (LF) (0.04–0.15 Hz) and High frequency (HF) (0.15–0.4) bands, which represent the influence of sympathetic and parasympathetic activity respectively. Also considered was the ratio of LF to HF (LF/HF) which represents the overall balance of the autonomic nervous system. Data were expressed using normalised units (nu), and all procedures were conducted in accordance with the recommendations of the Task Force of the European Society of Cardiology and The North American Society of Pacing and Electrophysiology^13^.

#### Non-linear analysis

##### Entropy

Entropy refers to system randomness, regularity and predictability and allows systems to be quantified by the amount of information within the signal. Approximate Entropy (ApEn) is commonly used to quantify the entropy of the system. ApEn examines a time series for similar segments and measures the likelihood that patterns that are close will remain close for subsequent incremental comparisons. A low value of ApEn reflects a high degree of regularity. However, ApEn has been criticized primarily due to its high dependence on the record length; with short records it is uniformly lower than expected^15^. As a result Richman and Moorman^15^ developed sample entropy (SampEn) which does not count self-matches and is less sensitive to data length. SampEn has been defined as the negative natural logarithm for conditional properties that a series of data points a certain distance apart, *m*, would repeat itself at *m* + 1 where self-matches are not included in calculating the probability^27^. A lower value of SampEn also indicates more regularity in the time series. The computation of sample entropy depends on two parameters; the embedding dimension *m* and the tolerance *r.* In the present study these were set as *m* = 2 and *r* = 0.2 SDNN. Formulae for calculating sample entropy (S1A) as well as a graphical representation (S1B) are available as supplementary information.

##### Detrended Fluctuation Analysis

Detrended fluctuation analysis (DFA) quantifies fractal like correlation properties of the time series and reveals short-range and long-range correlations. The DFA algorithm includes four steps: (1) removing the global mean and integrating the time series of a signal; (2) dividing the integrated signal into non-overlapping windows of the same chosen size *n*; (3) detrending the integrated signal in each window using polynomial functions to obtain residuals; and (4) calculating the root mean square of residuals in all windows as fluctuation amplitude F(*n*). The same four steps are repeated for different time scale *n* and plotted against window size on a log-log scale^28^. The scaling exponent DFA α indicates the slope of this line, which relates log(fluctuation) to log(window size). Formulae for calculating DFA (S2A) and an illustration of the double log plot (S2B) are available as supplementary information. Due to the length of the data series only short-term scaling exponent (α1) (calculated within range of 4–16 beats) was used for the analysis. The scaling exponent can be seen as a self-similarity parameter, which is characteristic of a fractal. Values of α around 1 indicate fractal like dynamics in healthy subjects; lower values indicate a loss of these dynamics.

##### Recurrence quantification analysis

Recurrence plots can be used to visualise the recurrence behaviour of the phase space trajectory of dynamical systems and have been applied successfully to the HR time series^16^. First a phase space trajectory is reconstructed from a time series by the time delay embedding *m.* Close states in the phase space can then be plotted as a recurrence plot according to the threshold *r.* A recurrence plot is a symmetrical matrix of zeros and ones [*N* − (*m* − 1)*τ*] × [*N* − (*m* − 1)*τ*] where *m* is the embedding dimension and *τ* the embedding lag. In the present study *m* *=* 10 and *r* *=* 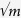 SDNN, where SD is the standard deviation of the RR time series. A sample recurrence plot is shown in Fig. 2. Recurrence quantification analysis (RQA)^29^ defines measures for diagonal segments in a recurrence plot including; recurrence rate (RPrec) which is the percent of plot filled with recurrent points and is the probability of recurrence, determinism (RPdet) which is the percent of recurrent points forming diagonal lines with a minimum of two adjacent points and measures predictability, the Shannon entropy of line length distribution (RPshen) and maximum line length (RPmax) which is inversely related to the largest positive Lyapunov exponent^30^ and is a measure of system divergence; a positive Lyapunov Exponent (LE) can be taken as a definition of chaos provided the system is known to be deterministic. Larger values of the LE indicate more complex behaviour.

On occasions (e.g. frequency domain measures) the calculated HR indices were not normally distributed. To correct for skewed distributions, a logarithmic transform was used before proceeding with stage 2 of the analysis.

### Stage 2 - Dimension reduction

A principal components factor analysis with varimax rotation was applied to the eleven HR indices that were calculated in stage 1. This analysis served two main purposes. Firstly, to reduce the large number of HR indices to a smaller number of meaningful components and secondly, to prevent problems of multicollinearity in the subsequent analysis. The results of this analysis are presented in Table 1. The analysis yielded three factors with eigenvalues greater than 1 which were saved as standardised individual factor scores and subsequently used for the analysis in stage 3.

The first factor, which explained 34% of the variance, encompassed the frequency domain indices (LF power, HF power and LH/HF ratio) as well as α1: a higher score on this factor indicates more vagal activity and more fractal-like dynamics (to aid interpretation it was multiplied by -1 so that a higher score was more positive). Due to the strong correlation with the frequency domain HR indices this factor is subsequently referred to as Frequency HRV. The second factor explained a further 34% of the variance and encompassed the RQA indices and sample entropy. This component reflected a complexity or irregularity dimension and is therefore referred to as HR complexity; again this factor was multiplied by −1 so that a higher score indicated greater complexity (i.e. more positive). The third factor, which explained 15% of the variance, included the time domain indices R-R and SDNN. This third component is referred to as Time HRV and a higher score on this component indicates a lower heart rate and greater variability (it was not multiplied by -1 because a higher score was already more positive). These factors were then entered into a multiple regression analysis to determine their relative association with aspects of cognition and mood (see below).

### Stage 3 – Regression analysis

To examine the relationship between the HRV components (derived in stage 2) and mood/cognition a series of hierarchical multiple regressions were conducted using SPSS version 20. To determine the moderating effect of sex, cross products were calculated for each HRV component X Sex and entered into the regression as additional predictors. Each HRV component was entered together with each HRV X Sex cross product. To reduce multicollinearity between each component and its respective cross product, variables were mean centred. Where interactions were significant, to elucidate any effect dependent on sex, conditional effects analysis was conducted using the SPSS macro PROCESS^31^. All regressions were carried out in a hierarchical fashion to determine the relative contribution of HR complexity after the other HRV factors had been considered. In the first model sex, frequency HRV (factor score 1), time HRV (factor score 3) and their respective cross products were entered. To ensure that any cognitive variation associated with HR complexity (factor score 2) and its sex cross product were not due to the other HRV factors, these were entered in the second step.

### Detection of Possible Outliers: Cook’s Distance

To detect possible outliers Cook’s Distance^32^ was calculated. The Cook’s Distance reflects the extent to which model residuals would change if a particular subject’s data (in multivariate space) were excluded from the estimated regression coefficient. Larger Cook’s Distance values indicate more influential subjects. The threshold for determining influential observations was set as 4/*N* in line with previous recommendations^33^. When certain cases had a Cook’s Distance that exceeded this threshold (the highest Cook’s Distance detected on any analysis was 0.3) those cases were excluded and the data re-analysed. On no occasion did this affect the outcome, and as there were no reasons to suspect these cases were unusual, the results reported included all cases.

### Control of the proportion of type 1 errors

Given the large number of associations tested in the present study there was a need to control the proportion of false positives among the set of rejected hypotheses. To do this Benjamini and Hochberg’s false discovery rate (FDR) was employed. The FDR was controlled at δ = 0.05 (Table S3).

## Results

Initially it was considered whether HRV differed in males and females at baseline. Independent t tests found no significant differences between males and females on any of the HRV indices (Table S4).

### Subjective measures

To measure subjective state participants completed the Profile of Mood States (POMS). The POMS measures six dimensions of mood: (1) Composed - Anxious; (2) Energetic - Tired; (3) Elated - Depressed; (4) Clear-headed - Confused; (5) Agreeable - Hostile; (6) Confident-Unsure. Participants also completed The Perceived Stress Scale (see methods).

Results for the individual mood dimensions and perceived stress are shown in Table 2. For Depressed/Elated, Clearheaded/Confused and Confident/Unsure the addition of HR complexity to the model explained a significantly greater proportion of the variable (R^2^ change = .21, F (2, 35) = 5.91, p < 0.006) for depression ratings; R^2^ change = .15, F (2, 33) = 3.858, p < 0.03 for ratings of confusion and R^2^ change = .12, F (2, 34) = 3.36, p < 0.04 for confidence ratings) although the only measure to survive the FDR correction was the rating of depression (Table S3). In fact, with ratings of depression the initial models containing only the time and frequency components did not reach significance (R^2^ = .15, F (5, 37) = 1.36, p = .260). Only when HR complexity was added did the model become significant (R^2^ = .36, F (7, 35) = 2.92, p < 0.01) explaining an extra 21%, and in total 36% of the variance. Similarly, with ratings of confusion model one was non-significant (R^2^ = .20, F (5, 35) = 1.84, p = .129). When HR complexity was added the model became significant (R^2^ = .35, F (7, 33) = 2.63, p < 0.02) explaining an extra 15%, and in total 35% of the variance of the ratings of confusion. Although with the ratings of confidence model one reached significance (R^2^ = .25, F (5, 36) = 2.278, p < .05), neither frequency nor time HRV were predictive; the only significant predictor was sex (β = −6.402, p < .005). When in model two HR complexity was added, it contributed significantly to the model (β = 3.656, p < .02) increasing the variance explained by 12%.

With ratings of confusion and ratings of depression there were significant interactions between sex and HR complexity (Table 2, Fig. 3), suggesting that sex moderates the relationship between HR complexity and these aspects of mood. Conditional effects analysis revealed that HR complexity significantly predicted depression in females (t(44) = 2.683, p < .001) but not males (t(44) = 0.298, p = .469). Similarly, HR complexity significantly predicted confusion in females (t(44) = 2.974, p < .02) but not males (t(44) = −0.278, p = .788). A higher HR complexity score was associated with a better mood in females.

HR complexity did not predict any other aspect of mood, rather with ratings of anxiety and perceived stress it was time HRV that was important (β = 2.67, p < .05 for anxiety; β = −2.91, p < .02 for perceived stress) (Table 2). A lower score on the time HRV component predicted more stress and anxiety. On these measures both the first and second models were significant, however the percentage of variance that was able to be explained was not increased by the addition of HR complexity (R^2^change = .05, F (2, 36) = 1.43 p = .252 for anxiety ratings; R^2^change = .05, F (2, 34) = 1.77, p = 0.185 for perceived stress). With perceived stress (β = −0.36, p < .04) there was also a Sex X Frequency HRV interaction (Table 2), however conditional effects analysis did not identify any effects that depended on sex (females; t(44) = −1.486, p = .145; males; t(44) = 2.219, p = .133). HRV did not predict ratings energy or agreeableness and with these dimensions none of the models were significant (Table 2).

### Cognitive performance

Results for each cognitive test are shown in Table 3. With focused attention inhibition (the ability to withhold responses to particular stimuli) the addition of HR complexity to the model increased the variance in behaviour explained increased by 23% (R^2^change = .23, F (2, 35) = 5.691, p < 0.007). The same pattern occurred with reaction times of the focused attention test; the addition of HR complexity explained an extra 11% of the variance in reaction times (R^2^change = .11, F (2, 37) = 3.093, p < 0.05) although this time the effect did not survive the FDR correction (Table S3). With both reaction times and inhibition (incorrectly responding to crosses) there were significant Sex X HR complexity interactions (Table 3), thus sex moderated the relationship between HR complexity and focused attention. Conditional effects analysis revealed that HR complexity significantly predicted reaction times in females (t(44) = −2.149, p < .03) but not males (t(44) = 0.448, p = .646; Fig. 4). Similarly, HR complexity predicted inhibition in females (t(44) = −2.279, p < .02) but not males (t(44) = 1.003, p = .321). A higher HR complexity score was associated with a better performance in females.

With decision times again only the second model, which contained HR complexity, was significant (R^2^ = .37, F(7, 39) = 3.130, p < .01). The addition of HR complexity increased the variance in decision times that could be explained by 21% (Table 3). The Sex X HR complexity interaction approached significance; HR complexity significantly predicted decision times in females (t(44) = −2.562, p < .01) but not males (t(44) = −1.192, p = .239). A higher HR complexity score was associated with a better performance in females.

There were no effects of HRV on working memory and neither the first nor the second models were significant (Table 3).

### Cortisol

Cortisol was measured at three time points: baseline, after relaxation, and after completing all of the tests. To evaluate whether HRV predicted cortisol levels a repeated measures ANCOVA was used. Cortisol was entered as a repeated measures factor (baseline, after relaxation, after cognitive tests) and sex as a between subjects factor. HRV components were entered as covariates and all main effects and 2-way interactions modelled. There was a main effect of measurement (i.e. baseline, after relaxation, after the tests) (F(2, 70) = 5.922, p  < 0.004); cortisol levels were lower once testing had finished (baseline; 0.20(0.11), after relaxation; 0.18 (0.10) and after cognitive tests; 0.15(0.09)). There was a significant time HRV X Cortisol measurement interaction (F(2, 70) = 3.992, p < 0.02) such that time HRV predicted a change in cortisol measurement from baseline to after relaxation (r = −.307, p < .05); a higher time HRV predicted a greater decline in cortisol from baseline to after relaxation. Frequency HRV did not predict cortisol levels, however, the interaction Sex X HR complexity reached significance (F(1, 35) = 7.069, p < 0.01). Conditional effects analysis revealed that the effect of HR complexity approached significance in females (t(44) = −1.788 p  < 0.07) but not in males (t(44) = 1.544, p = 138); females with a higher complexity score tended to have lower cortisol levels.

- With ratings of depression, the speed of focused attention and decision times and inhibition, adding HR complexity to the model significantly increased the percentage of the variance that could be explained.
- HR complexity was independently associated with behaviour after accounting for any variation associated with the other HRV components.
- The effects of HR complexity were dependent on sex; HR complexity was related to mood, cognition and cortisol levels in females but not males.
- Whereas HR complexity was related to hedonic aspects of mood (e.g ratings of depression) and cognition, linear HRV was most strongly associated with perceived stress and anxiety.

## Discussion

The present study determined the association between non-linear HRV indices, cognition and mood. It was found that on a number of occasions HR complexity significantly increased the percentage of variance in behaviour that could be explained. HR complexity was independently associated with ratings of mood (Fig. 3), focused attention reaction times (Fig. 4), inhibition, and decision times. Importantly, these effects remained after any variance associated with frequency and time HRV indices had been considered. Thus HR complexity was able to capture additional information to that obtained using traditional HRV measurements. Previously it had been reported that HR complexity indices were able to identify considerable changes in autonomic regulation in those with clinical depression whereas linear HRV did not^18^. Similarly, Bornas *et al.*^34^ found that whereas frequency domain HRV did not predict the treatment outcome in those afraid of flying, the addition of HRV SampEn to the regression model increased its predictive power by an additional 18%. These studies suggested that HR complexity may not only capture additional information on top of that obtained with traditional HRV, but in some instances may be the essential component. Indeed, the present study supports this notion; only when HR complexity was added to the model was it significantly related to ratings of depression (Fig. 3), focused attention reaction times, decision times and salivary cortisol – although frequency and time HRV were associated with perceived stress and anxiety.

A second aim was to establish whether sex moderated the relationship between linear and nonlinear HRV indices and behaviour. Previous evidence suggests that females exhibit greater parasympathetic activity at rest while greater sympathetic activity was found in males^35^. The existence of a sex difference in HRV suggested a possible role for gonadal hormones on vagal activity, a possibility which is supported by evidence from studies using rats. For example, the heart rate lowering effect of vagal stimulation is reduced by ovariectomy^35^. Similarly, studies of human females indicated higher HF-power, and thus vagal tone, during elevated oestradiol phases of the menstrual cycle^36^; although others report no differences across the cycle^37^. With regards to nonlinear HR dynamics, it was found that both entropy and the fractal nature of HRV were altered during the regular menstrual cycle of women; changes that were positively associated with the ratio of oestradiol-17 to progesterone in the blood^9^.

However, the present study did not detect sex differences in HRV at baseline (Table S3); rather it was the relationship between HRV at baseline and subsequent behaviour that depended upon sex. Whereas time and frequency HRV were associated with behaviour in males and females, HR complexity was more strongly associated with behaviour in females (Table 3). Sex differences in brain structure and function have long been recognised^38^, but it is noteworthy that sex differences in the complexity of EEG signals have recently been found with females displaying a more fractal EEG activity^39^ and higher entropy^40^ than males. It is also interesting that a positive correlation between cerebral complexity and cognitive performance has also been reported^41^,^43^), although it remains to be determined if these relationships differ according to sex.

It has been suggested that gonadal hormones in the central nervous system (CNS), including regions relevant for the functioning of the autonomic nervous system, may play a role in sex related HRV differences^44^. Several studies have investigated the brain circuitry correlates of ANS regulation and generally report activation of hypothalamic nuclei, brainstem regions, amygdala, hippocampus, and the frontal and cingulate cortices^4^. Interestingly, gonadal hormone signalling and their functions are highly sexually dimorphic in many of these regions^42^, supporting their possible role in modulating HRV. In support of this view, a recent study found that hypoactivation of the hypothalamus, amygdala, hippocampus, cingulate cortex and orbitofrontal cortex were associated with lower parasympathetic activity in depressed women; associations that were attenuated when differences in oestradiol and progesterone were considered^43^. Yao *et al.*^45^, in a large meta-analysis of 26 datasets, examined the entropy of resting state BOLD signals depending on sex and age. Intriguingly, a crossover occurred at around 50 years of age; around the age that women experience the menopause. Males over the age of 50 had higher entropy, whereas below this age the roles were reversed. Cerebral entropy has been shown to correlate with HR entropy^46^; however, it is not known whether a similar pattern of findings to those of Yao *et al*^45^ would be observed with nonlinear HRV indices.

Although previous research has found connections between HRV and subjects’ cortisol response to cognitive tasks^47^, to our knowledge this is the first study to relate HR complexity to cortisol levels. It was interesting that only HR complexity predicted cortisol levels, and that this effect was confined to females. Sex differences in cortisol response have been recognised, which have again been suggested to reflect sexual dimorphic aspects of brain functioning and the role of circulating sex steroids^48^. A limitation of the present study is that sex hormone levels were not recorded. Thus it cannot be established whether sex hormone levels played a role in the present findings, although this possibility needs to be considered. In addition, the majority of females in the present study were taking some form of contraceptive pill (n = 20/24) such that natural hormonal variation would be supressed. Further research is needed to establish the connections between the hypothalamic gonadal axis, hypothalamic pituitary axis, nonlinear HRV, brain functioning and cognition.

Although the physiological underpinnings of nonlinear HRV remain elusive, there is clear evidence that these indices present a promising way of quantifying heart rate dynamics and using them as markers of the functioning of the CNS. Earlier studies have attempted to clarify the contribution of autonomic nervous system (ANS) activity to HR complexity using pharmacological intervention. Perkiomaki *et al.*^49^ measured linear (SDNN, RMSSD, LF power, HF power) and nonlinear (short-term scaling exponent (α1), approximate entropy (ApEn)) HRV for 5 minute periods before and after the intravenous injection of 0.6 mg of atropine (parasympathetic antagonist). Whereas α1 increased significantly after atropine injection and correlated significantly with several linear HRV indices, ApEn did not. This suggested that vagal tone has a significant contribution to the fractal nature of the HR time series but is not a major determinant of its entropy. The present study supports this notion; principle component analysis revealed that α1 loaded heavily onto the same factor as all the frequency domain indices; however SampEn and the recurrent plot (RP) indices loaded together onto a separate factor (Table 1). Therefore, it appears that both HR entropy and RP analyses are able to capture additional information contained with the HR time series that is not attributable to ANS activity. Given the strong relationship between these indices and cognition in females, the hypothalamic gonadal axis may be part of the physiological processes underlying the information contained in HR entropy.

It remains to be determined what cerebral processes are captured by HR complexity. Normal physiological functioning requires the integration of intricate networks of control systems, feedback loops and other regulatory mechanisms, to enable an organism to perform simultaneously, many and varied activities. A dominant theory has been that complexity describes the dynamics of these integrated physiological processes. With increased complexity a system has greater adaptability; a reduction in complexity, as a result of aging or disease, would result in an inflexible system that is more easily compromised^50^. Indeed neurophysiological evidence has shown that as a mental task becomes more difficult EEG entropy is decreased^51^, suggesting a lack of spare cognitive capacity when a more difficult task is performed. Sokunbi *et al.*^52^ studied the relationship between BOLD signal entropy, childhood intelligence and current cognitive ability of older adults. Although cerebral entropy was associated with current cognitive ability it was not related to childhood intelligence. Similarly, resting state BOLD signal entropy was associated with the level of cognitive impairment in those with dementia^53^ and correlates with cognition in those with traumatic brain injury^54^. Interestingly, Yang *et al.*^41^ examined the link between the entropy of resting-state BOLD activity and participant’s performance on a battery of cognitive tests. Not only was there an age-related loss of complexity in multiple brain regions but, in line with the present findings, the degree of complexity was positively correlated with attention, memory and verbal fluency, whereas traditional measures of variability (SD of BOLD signals) were not. A speculative suggestion is that physiological complexity, of which HR complexity is one facet, may be an indicator of ‘cognitive reserve’ and useful in the identification of those at risk of cognitive decline. Although HR complexity declined with age^55^, to date the link between HR complexity and ‘cognitive reserve’ has not been considered and research is needed to determine whether HR complexity predicts cognitive decline.

Whilst the present study raises some intriguing questions it is not without its limitations. There is a need to replicate the present findings in a larger sample. In addition, although the majority of studies of HRV have not measured respiration it is potentially a confounding variable, particularly with frequency domain measures. With a low rate of respiration parasympathetic activity moves into a lower frequency range that can overlap with the range that defines sympathetic activity^56^. However, it has been suggested that nonlinear HRV measures are not affected by the rate of breathing^57^,^58^ demonstrating that nonlinear measures of HRV did not result from a nonlinear respiration input. In fact, Lund *et al.*^59^ justified not monitoring breathing by the stating that nonlinear HRV were not susceptible to the nature of breathing. However, although there is no reason to believe that respiration varied systematically such that it would have biased the present findings, it is a question to be addressed in future studies as some have suggested that non-linear measures are influenced by respiration^60^.

## Conclusion

In conclusion, many physiological processes are known to be nonlinear, including HRV and brain functioning^6^, such that nonlinear rather than linear HR indices more successfully predict complex behaviour. The present study found that HR complexity (SampEn, RP analysis) was independently associated with aspects of cognition and mood and in some instances were able to explain behaviour when traditional linear methods could not. In addition, the relationship between HR complexity, cognition and mood was highly dependent on sex, thus these indices may be particularly useful when explaining the behaviour of females. Future research should consider the influence of the hypothalamic gonadal axis on the modulation of HRV, in particular HR entropy and recurrence quantification analysis indices. In addition, given the possible links between physiological complexity and ‘cognitive reserve’, further research should consider the potential usefulness of HR complexity in predicting cognitive decline and other neuropsychological disorders.

## Methodology

### Participants

Twenty one males and twenty four female undergraduates gave their written informed consent (sample characteristics are shown in Table 4). Participants were excluded if they had any health complaint that would affect cardiovascular functioning such as diabetes or hypertension. Similarly, anyone with a neuropsychological illness was also excluded as were those taking medication (with the exception of the contraceptive pill). All participants were non-smokers and were asked to refrain from drinking alcohol for at least 24 hours before the start of the study. In addition, participants were asked to fast (except water) and avoid any caffeinated beverages for at least two hours before the start of the study.

### Procedure

**Figure 1:**
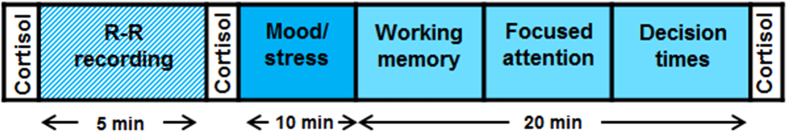
Schematic representation of the experimental procedure.

**Figure 2:**
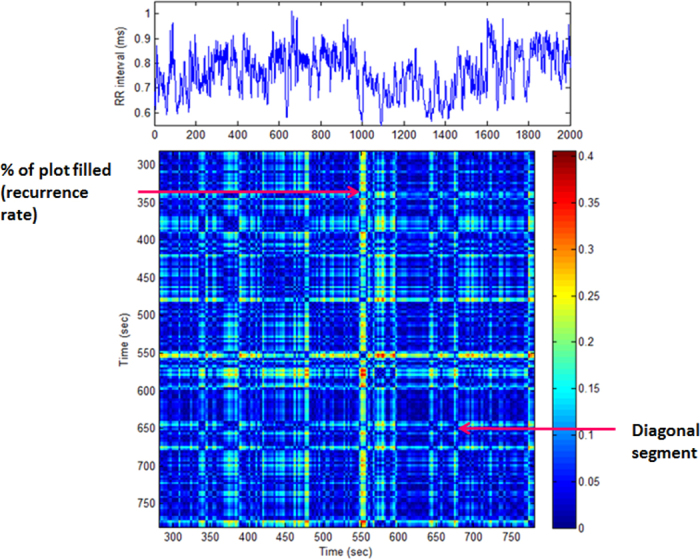
Sample recurrence plot matrix for HRV time series. Data shown are taken from a female participant in the present study for illustrative purposes.

**Figure 3:**
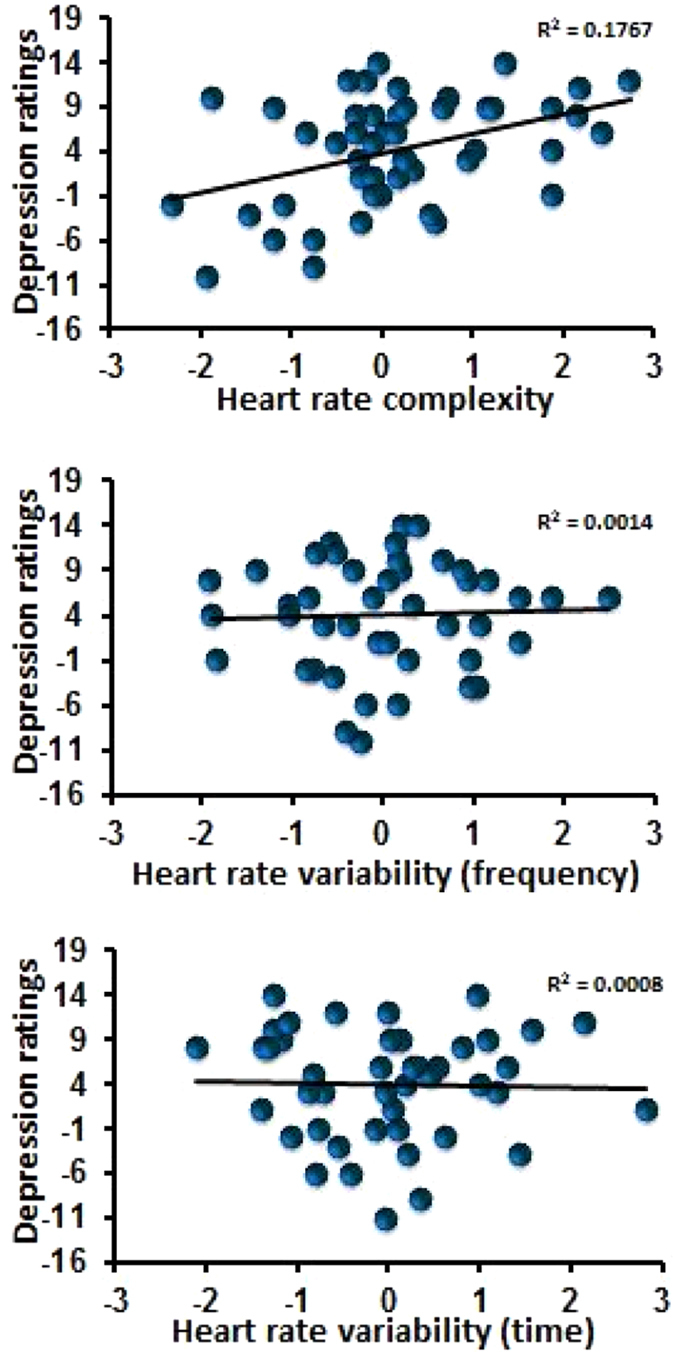
The associations between HR complexity and HR variability (frequency and time domain) and ratings of depression. HR complexity was related to ratings of depression (β = 4.603, p < 0.002) however HR variability was not (Frequency: β = 0.348, p = 0.08, Time β = −.055, p = .795).

**Figure 4:**
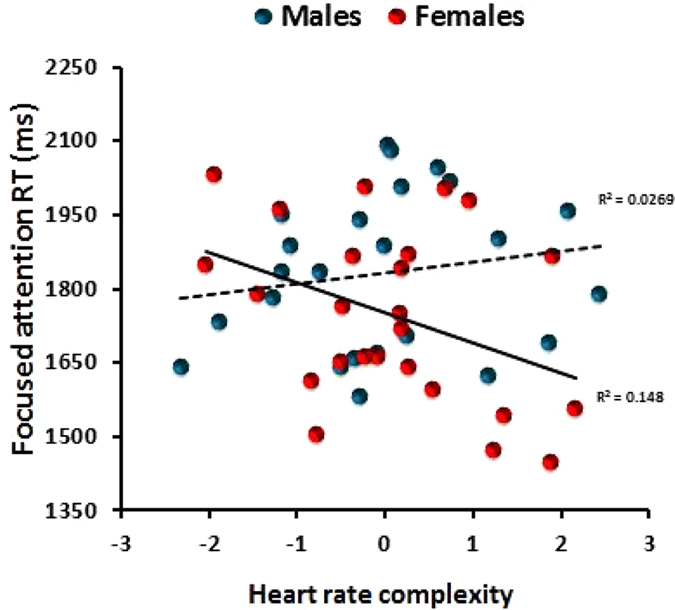
The relationship between HR complexity and focused attention reaction times depending on gender. A higher HR complexity significantly predicted quicker reaction times in females (t(44) = −2.149, p < .03) but not males (t(44) = 0.448, p = .646).

The procedure is outlined in Fig. 1. Upon arrival at the laboratory participants were fitted with a RS800 Polar heart rate monitor electrode transmitter belt (T61) using conductive gel as recommended by the manufacturer. Interbeat interval measurements were collected using a Polar RS800 HR monitor set to R-R interval mode (Polar Electro, Kempele, Finland) at a sampling rate of 1000 Hz. This instrument has been previously validated for the accurate measurement of R-R intervals and for analysing Heart Rate Variability (HRV)^61^. Participants were seated comfortably and asked to relax for five minutes while listening to calming music (Tranquillity of Baroque, Warner Music). The HR time series was recorded during this five minute relaxation period. After the relaxation period participants completed a number of questionnaires about their recent mood and stress levels and a cognitive test battery (outlined below). The procedure was approved by Swansea University ethics committee (reference number: 07.25.2013.1) and carried out in accordance with the Declaration of Helsinki - Ethical Principles for Medical Research Involving Human Subjects.

### Cortisol

Salivary cortisol (ug/dl) levels were monitored on arrival at the laboratory (baseline), after relaxation and again after the cognitive tests had been completed. Testing was carried out in the afternoon to avoid the high levels of cortisol that are found in the first five hours after waking. Samples were collected using Salimetrics SalivaBio Oral Swab and Swab Storage Tube and immediately frozen below −20 °C. Analysis was carried out using an immunoassay supplied by Salimetrics Europe Ltd, Suffolk, UK.

### Cognition

#### Working memory – Serial sevens

Serial Sevens involved presenting a participant with a starting number from which they must serially subtract 7, and is considered a sensitive measure of working memory. A computerised version of the Serial Sevens test was implemented. A randomly generated starting number between 800 and 999 was displayed on a monitor for two seconds. Participants were required to indicate by pressing one of two keys, that corresponded to yes or no, whether a second number was or was not exactly seven less. Two seconds after pressing the button the next trial started and a total of twenty-eight trials were completed. The test was scored as the average of the time taken, in milliseconds, to perform a subtraction.

### Focused attention - Arrow Flankers Test

A modified version of the Eriksen and Eriksen^62^ flanker task was used to measure focused attention. The Arrow Flankers test measures the ability to direct attention and ignore peripheral information. Participants were required to indicate whether the middle arrow was pointing to the right or left by pressing the corresponding arrow on the keyboard. Either side of the central arrow were distractors. The flanking pairs of symbols could be squares (□□ <□□), crosses (xx < xx), congruent arrows (pointing in the same direction (≫ > ≫)) or incongruent arrows (pointing in the opposite direction (≫ < ≫)). Participants had to respond as quickly as possible to squares, congruent arrows and incongruent arrows but were instructed to inhibit their response when the peripheral information was crosses. A stimulus remained on screen for 1.8 seconds or until the key press was registered. There was a randomly varying inter-stimulus interval of between 1 and 3 seconds, on average 2 seconds. Seventy stimuli were presented of which 10 were crosses with the remainder occurring pseudo-randomly with congruent, incongruent or neutral (squares) stimuli appearing on twenty occasions. The test was scored as the average response time in milliseconds. In addition, the number of incorrect presses was recorded as a measure of the ability to inhibit responses.

### Simple and choice decision times

The reaction time procedure was based on that of Jensen^63^. Eight lamps were arranged in a semicircle 5.5 inches from a central button (the home key). The index finger was placed on the home key. Within one to two seconds an auditory warning signal sounded and after a random interval of one to four seconds one of the lamps illuminated. The task was to extinguish the light by depressing a button directly below the lamp, using the finger initially on the home key. All participants completed a practice session of 20 trials utilising all eight lamps. Participants were then told that they would complete four blocks or 20 trials and that they should respond as quickly as possible. Simple reaction times were measured for 20 trials using one lamp. Choice reaction times were then measured over three sets of 20 trials when one of 2, 4 or 8 lamps could be potentially illuminated. Decision times, the time in milliseconds taken to lift the finger from the home key, were analysed. Decision times of less than 150 ms were discarded as outliers, as it has been argued that physiological limits prevent shorter DTs^61^. Decision times over 999 ms were also discarded and replaced with an additional trial. In addition, all decision times exceeding three standard deviations above the subject’s mean DT were also discarded^61^.

## Mood

### Profile of Mood States

The Profile of Mood States Bi-Polar Form (POMS)^64^ is a 72-item self-report questionnaire that measures six dimensions of mood: (1) Composed - Anxious; (2) Energetic - Tired; (3) Elated - Depressed; (4) Clear-headed - Confused; (5) Agreeable - Hostile; (6) Confident-Unsure. Participants were presented with a list of words or phrases and had to rate on a scale of 0–3 (0 ‘not at all’, 3 ‘a lot like this’) how much they had felt like this in the past week including today. There are twelve words for each mood dimension – six positive and six negative.

### Perceived Stress Scale

The Perceived Stress Scale^65^ is a 10-item self-report questionnaire that assesses the degree to which situations in one’s life are perceived as stressful. The participants were required to answer questions about the extent to which they have had stressful thoughts and feeling during the last week, for example, “In the last week, how often have you been upset because of something that happened unexpectedly?” The participant responds on a 5-point scale (ranging from 0 = Never - 4 = Very Often). One overall score was produced by summing across all items.

## Additional Information

**How to cite this article**: Young, H. and Benton, D. We should be using nonlinear indices when relating heart-rate dynamics to cognition and mood. *Sci. Rep.* **5**, 16619; doi: 10.1038/srep16619 (2015).

## Supplementary Material

Supplementary Information

## Figures and Tables

**Table 1:** Loading matrix for the Principle Component Analysis.

INDICES	FACTORS		
	Frequency HRV	HR complexity	Time HRV
R-R (ms)	−0.243	−0.182	**0.764**
SDNN (ms)	−0.052	0.254	**0.869**
LF power (nu)	**0.941**	0.250	−0.129
HF power (nu)	−**0.941**	−0.252	0.127
LF/HF ratio	**0.942**	0.121	−0.059
RPmax (beats)	0.380	**0.503**	−0.429
RPrec (%)	0.105	**0.935**	−0.023
RPdet (%)	0.380	**0.858**	−0.113
RPshen	0.164	0.**939**	0.026
α1	**−0.837**	0.250	−0.316
SampEn	−0.204	**−0.817**	−0.085

Correlations between each HR index and the factors are shown. The analysis gave rise to three components. Frequency domain HR indices loaded heavily onto component one while the nonlinear HR indices and time domain indices loaded onto components two and three respectively. R-R interval - The mean of RR intervals, SDNN – Standard deviation of RR intervals, LF – Low frequency, HF – High frequency, RPmax - Maximum line length of diagonal lines in recurrence plot, RPrec - Recurrence rate (percentage of recurrence points in recurrence plot), RPdet - Determinism (percentage of recurrence points which form diagonal lines in recurrence plot), RPshen - Shannon entropy of diagonal line lengths’ probability distribution, α1 - Short-term fluctuations of detrended fluctuation analysis, SampEn - Sample entropy.

**Table 2:** Regression analyses evaluating the contribution of Time, Frequency and HR complexity to mood and perceived stress.

DV	IV	*β*	R^2^	F	*p*
Elated/Depressed	STEP 1		0.15	1.36	0.260
	**STEP 2**		0.36	2.92	0.016
	Gender	−0.206			0.191
	Frequency HRV	0.348			0.082
	Frequency X gender	−0.069			0.723
	Time HRV	−0.055			0.795
	Time X gender	0.205			0.336
	**STEP 2**		0.36	2.92	0.016
	Gender	−2.386			0.191
	Frequency HRV	2.367			0.042
	Frequency X gender	−0.883			0.628
	Time HRV	0.919			0.476
	Time X gender	0.473			0.802
	Complexity HRV	4.603			0.002
	Complexity X gender	−3.847			0.044
	**Change statistics**	**R^2^change = 0.21, F (2, 35) = 5.91, p < 0.006**			
Anxious/Composed	**STEP 1**		0.23	2.34	0.060
	Gender	−2.270			0.233
	Frequency HRV	2.017			0.098
	Frequency X gender	−0.979			0.611
	Time HRV	2.023			0.126
	Time X gender	0.511			0.789
	**STEP 2**		0.29	2.11	0.011
	Gender	−2.152			0.254
	Frequency HRV	2.071			0.087
	Frequency X gender	−1.080			0.572
	Time HRV	2.671			0.053
	Time X gender	−0.288			0.884
	Complexity HRV	2.335			0.110
	Complexity X gender	−1.805			0.350
	**Change statistics**	R^2^change = 0.05, F (2, 36) = 1.43 p = 0.252			
Clearheaded/confused	**STEP 1**		0.20	1.846	0.129
	Gender	−1.982			0.305
	Frequency HRV	2.038			0.079
	Frequency X gender	−5.131			0.025
	Time HRV	−0.152			0.903
	Time X gender	1.217			0.520
	**STEP 2**		0.35	2.636	0.028
	Gender	−1.879			0.296
	Frequency HRV	2.107			0.052
	Frequency X gender	−4.964			0.021
	Time HRV	0.702			0.561
	Time X gender	0.684			0.710
	Complexity HRV	3.442			0.012
	Complexity X gender	−4.325			0.**017**
	**Change statistics**	R^2^ = 0.15, F (2, 33) = 3.858 p = 0.031			
Agreeable/Hostile	**STEP 1**		0.098	0.807	0.552
	Gender	−1.717			0.290
	Frequency HRV	−0.964			0.341
	Frequency X gender	1.437			0.374
	Time HRV	−0.494			0.643
	Time X gender	1.726			0.282
	**STEP 2**		0.134	0.771	0.615
	Gender	−1.786			0.275
	Frequency HRV	−0.980			0.338
	Frequency X gender	1.446			0.375
	Time HRV	−0.586			0.599
	Time X gender	2.484			0.158
	Complexity HRV	−0.381			0.753
	Complexity X gender	−1.120			0.531
	**Change statistics**	R^2^ = 0.03, F (2, 35) = 0.547 p = 0.497			
Confident/Unsure	**STEP 1**		0.256	2.278	0.050
	Gender	−6.402			0.005
	Frequency HRV	1.722			0.183
	Frequency X gender	−1.847			0.385
	Time HRV	1.291			0.369
	Time X gender	−1.713			0.423
	**STEP 2**		0.379	2.965	0.015
	Gender	−6.634			0.002
	Frequency HRV	1.835			0.134
	Frequency X gender	−2.159			0.285
	Time HRV	2.322			0.107
	Time X gender	−3.227			0.133
	Complexity HRV	3.657			0.021
	Complexity X gender	−2.405			0.241
	**Change statistics**	R^2^change = 0.12, F (2, 34) = 3.36, p < 0.041			
Energetic/Tired	**STEP 1**		0.112	1.148	0.440
	Gender	0.305			0.057
	Frequency HRV	−0.053			0.786
	Frequency X gender	0.146			0.455
	Time HRV	−0.126			0.553
	Time X gender	0.130			0.540
	**STEP 2**		0.165	1.045	0.417
	Gender	0.293			0.066
	Frequency HRV	−0.052			0.789
	Frequency X gender	0.151			0.437
	Time HRV	−0.043			0.845
	Time X gender	0.099			0.649
	Complexity HRV	0.330			0.165
	Complexity X gender	−0.337			0.157
	**Change statistics**	R^2^change = 0.05, F (2, 39) = 1.174, p = 0.320			
Perceived stress	**STEP 1**		0.36	4.002	0.006
	Gender	4.575			0.009
	Frequency HRV	−2.065			0.050
	Frequency X gender	4.306			0.012
	Time HRV	−2.218			0.067
	Time X gender	0.572			0.735
	**STEP 2**		0.42	3.460	0.007
	Gender	4.467			0.010
	Frequency HRV	−2.123			0.040
	Frequency X gender	4.339			0.011
	Time HRV	−2.915			0.021
	Time X gender	1.169			0.500
	Complexity HRV	−2.261			0.072
	Complexity X gender	2.583			0.121
	**Change statistics**	R^2^change = 0.06, F (2, 33) = 1.70, p = 0.198			

Only effects that survived the FDR correction are highlighted.

**Table 3:** Regression analyses evaluating the contribution of Time, Frequency and HR complexity to cognition.

DV	IV	*β*	R^2^	F	*p*
Working memory	**STEP 1**		0.091	0.863	0.515
	Gender	−0.078			0.624
	Frequency HRV	−0.184			0.353
	Frequency X gender	−0.127			0.519
	Time HRV	0.078			0.717
	Time X gender	−0.163			0.447
	**STEP 2**		0.127	0.830	0.562
	Gender	−0.075			0.639
	Frequency HRV	−0.183			0.358
	Frequency X gender	−0.137			0.490
	Time HRV	0.129			0.567
	Time X gender	−0.238			0.291
	Complexity HRV	0.201			0.405
	Complexity X gender	−0.004			0.988
	**Change statistics**	R^2^change = 0.03, F (2, 39) = 0.769, p = 0.471			
Focused attention reaction times	**STEP 1**		0.19	1.889	0.117
	Gender	−78.717			0.130
	Frequency HRV	−23.061			0.478
	Frequency X gender	9.975			0.849
	Time HRV	65.476			0.070
	Time X gender	−133.621			0.014
	**STEP 2**		0.31	2.386	0.057
	Gender	0.078			0.078
	Frequency HRV	0.456			0.456
	Frequency X gender	0.922			0.922
	Time HRV	0.168			0.168
	Time X gender	0.010			0.**010**
	Complexity HRV	0.089			0.089
	Complexity X gender	.018			**0.018**
	**Change statistics**	R^2^change = 0.11, F (2, 37) = 3.093, p < 0.05			
Focused attention inhibition	**STEP 1**		0.03	0.203	0.959
	Gender	−0.446			0.575
	Frequency HRV	−0.194			0.701
	Frequency X gender	0.361			0.657
	Time HRV	−0.146			0.789
	Time X gender	−0.225			0.807
	**STEP 2**		0.26	1.807	0.117
	Gender	−0.136			0.850
	Frequency HRV	−0.178			0.692
	Frequency X gender	0.346			0.634
	Time HRV	−0.555			0.278
	Time X gender	0.307			0.715
	Complexity HRV	−1.597			0.**005**
	Complexity X gender	2.506			0.**003**
	**Change statistics**	**R^2^change = 0.23, F (2, 35) = 5.691, p < 0.007**			
Decision times	**STEP 1**		0.15	1.417	0.240
	Gender	52.256			0.042
	Frequency HRV	−23.399			0.145
	Frequency X gender	24.971			0.334
	Time HRV	−8.687			0.616
	Time X gender	16.018			0.531
	**STEP 2**		0.37	3.130	0.011
	Gender	47.627			0.038
	Frequency HRV	−23.404			0.101
	Frequency X gender	26.214			0.253
	Time HRV	−23.728			0.142
	Time X gender	35.153			0.140
	Complexity HRV	−58.373			0.**001**
	Complexity X gender	44.063			0.060
	**Change statistics**	**R^2^change = 0.21, F (2, 42) = 6.425, p < 0.004**			

Only effects that survived the FDA correction are reported.

**Table 4:** Descriptive data for males and females.

	MALES	FEMALES
N	21	24
Age	21.2(2.1)	22.2(3.8)
BMI	23.4(3.4)	23.5(4.5)

Data are mean (SD).

## References

[b1] Benarroch E. E. The central autonomic network: functional organization, dysfunction, and perspective. Mayo Clin. Proc. 68, 988–1001 (1993). 8412366 10.1016/s0025-6196(12)62272-1

[b2] Thayer J. F. & Lane R. D. Claude Bernard and the heart-brain connection: further elaboration of a model of neurovisceral integration. Neurosci. Biobehav. Rev. 33, 81–88 (2009). 18771686 10.1016/j.neubiorev.2008.08.004

[b3] Hansen A. L., Johnsen B. H. & Thayer J. F. Vagal influence on working memory and attention. Int. J. Psychophysiol. 48, 263–274 (2003). 12798986 10.1016/s0167-8760(03)00073-4

[b4] Thayer J. F., Åhs F., Fredrikson M., Sollers J. J. III & Wager T. D. A meta-analysis of heart rate variability and neuroimaging studies: implications for heart rate variability as a marker of stress and health. Neurosci. Biobehav. Rev. 36, 747–756 (2012). 22178086 10.1016/j.neubiorev.2011.11.009

[b5] Perkiömäki J. S. Heart rate variability and non-linear dynamics in risk stratification. Front. Physiol. 2, 81 (2011). 22084633 10.3389/fphys.2011.00081 PMC3210967

[b6] Mattei T. A. Unveiling complexity: non-linear and fractal analysis in neuroscience and cognitive psychology. Front. Comput. Neurosci. 8, 17 (2014). 24600384 10.3389/fncom.2014.00017 PMC3930866

[b7] Sztajzel J. Heart rate variability: a noninvasive electrocardiographic method to measure the autonomic nervous system. Swiss Med. Weekly 134, 514–522 (2004). 10.4414/smw.2004.10321 15517504

[b8] Cui J. *et al.* Effects of heat stress on thermoregulatory responses in congestive heart failure patients. Circulation 112, 2286–2292 (2005). 16216975 10.1161/CIRCULATIONAHA.105.540773

[b9] Bai X., Li J., Zhou L. & Li X. Influence of the menstrual cycle on nonlinear properties of heart rate variability in young women. Am. J. Physiol. - Heart Circulat. Physiol. 297, H765–H774 (2009). 10.1152/ajpheart.01283.2008 19465541

[b10] Rongen G. A. *et al.* Effect of adenosine on heart rate variability in humans. Clin. Sci. 96, 597–604 (1999). 10334965

[b11] Massin M. M., Maeyns K., Withofs N., Ravet F. & Gérard P. Circadian rhythm of heart rate and heart rate variability. Arch. Dis. Child. 83, 179–182 (2000). 10906034 10.1136/adc.83.2.179 PMC1718415

[b12] Alderman B. L. & Olson R. L. The relation of aerobic fitness to cognitive control and heart rate variability: A neurovisceral integration study. Biol. Psychol. 99, 26–33 (2014). 24560874 10.1016/j.biopsycho.2014.02.007

[b13] Camm A. J. *et al.* Heart rate variability: standards of measurement, physiological interpretation and clinical use. Task Force of the European Society of Cardiology and the North American Society of Pacing and Electrophysiology. Circulation 93, 1043–1065 (1996). 8598068

[b14] PerkiöMäKi J. S., Mäkikallio T. H. & Huikuri H. V. Fractal and complexity measures of heart rate variability. Clin. Exp. Hyperten. 27, 149–158 (2005). 15835377

[b15] Richman J. S. & Moorman J. R. Physiological time-series analysis using approximate entropy and sample entropy. Am. J. Physiol. Heart Circ. Physiol. 278, H2039–H2049 (2000). 10843903 10.1152/ajpheart.2000.278.6.H2039

[b16] Webber C. L. Jr & Zbilut J. P. Dynamical assessment of physiological systems and states using recurrence plot strategies. J. Appl. Physiol. 76, 965–973 (1994). 8175612 10.1152/jappl.1994.76.2.965

[b17] Voss A. *et al.* The application of methods of non-linear dynamics for the improved and predictive recognition of patients threatened by sudden cardiac death. Cardiovasc. Res. 31, 419–433 (1996). 8681329

[b18] Voss A., Schulz S., Koschke M. & Bar K. J. Linear and nonlinear analysis of autonomic regulation in depressed patients. Conf. Proc. IEEE Eng. Med. Biol. Soc. 2653–2666 (2008). 19163250 10.1109/IEMBS.2008.4649747

[b19] Bar K. J. *et al.* A. Non-linear complexity ¨measures of heart rate variability in acute schizophrenia. Clin. Neurophysiol. 118, 2009–2015 (2007). 17646130 10.1016/j.clinph.2007.06.012

[b20] Swaab D. F. Sexual differentiation of the brain and behavior. Best Pract. Res. Clin. Endocrinol. Metabol. 21, 431–444 (2007). 10.1016/j.beem.2007.04.003 17875490

[b21] Sandu A. L., Specht K., Beneventi H., Lundervold A. & Hugdahl K. Sex-differences in grey–white matter structure in normal-reading and dyslexic adolescents. Neurosci. Lett. 438, 80–84 (2008). 18456405 10.1016/j.neulet.2008.04.022

[b22] Gur R. C., Gunning-Dixon F., Bilker W. B. & Gur R. E. Sex differences in temporo-limbic and frontal brain volumes of healthy adults. Cereb. Cortex. 12, 998–1003 (2002). 12183399 10.1093/cercor/12.9.998

[b23] Saleem S., Hussain M. M., Majeed S. M. I. & Khan M. A. Gender differences of heart rate variability in healthy volunteers. J. Pak. Med. Assoc. 62, 422–425 (2012). 22755301

[b24] Voss A., Schroeder R., Heitmann A., Peters A. & Perz S. Short-term heart rate variability—influence of gender and age in healthy subjects. PloS one. 10, 3, doi: 10.1371/journal.pone.0118308 (2015). PMC4378923 25822720

[b25] Gillie B. L. & Thayer J. F. Individual differences in resting heart rate variability and cognitive control in posttraumatic stress disorder. Front. Psychol. 5, 758 (2014). 25076929 10.3389/fpsyg.2014.00758 PMC4097943

[b26] Schulz S., Koschke M., Bär K. J. & Voss A. The altered complexity of cardiovascular regulation in depressed patients. Physiol. Measure. 31, 303–321 (2010). 10.1088/0967-3334/31/3/003 20086275

[b27] Yentes J. M. *et al.* The appropriate use of approximate entropy and sample entropy with short data sets. Ann. Biomed. Eng. 41, 349–365 (2013). 23064819 10.1007/s10439-012-0668-3 PMC6549512

[b28] Peng C. K., Havlin S., Stanley H. E. & Goldberger A. L. Quantification of scaling exponents and crossover phenomena in nonstationary heartbeat time series. Chaos 5, 82–87 (1995). 11538314 10.1063/1.166141

[b29] Zbilut J. P., Thomasson N. & Webber C. L. Recurrence quantification analysis as a tool for nonlinear exploration of nonstationary cardiac signals. Med. Eng. Phys. 24, 53–60 (2002). 11891140 10.1016/s1350-4533(01)00112-6

[b30] Eckmann J. P., Kamphorst S. O. & Ruelle D. Recurrence plots of dynamical systems. Europhys. Lett. 4, 973–7 (1987).

[b31] Hayes A. F. Introduction to mediation, moderation, and conditional process analysis: A regression-based approach (ed. Hayes A. F. ) 149–157 (Guilford Press, 2013).

[b32] Cook R. D. Detection of influential observation in linear regression. Technometrics 19, 15–18 (1977).

[b33] Bollen K. A. & Jackman R. W. Regression diagnostics: an expository treatment of outliers and influential cases. In Modern Methods of Data Analysis (eds. Fox John & Long J. Scott ) (Newbury Park, CA, Sage 1990).

[b34] Bornas X. *et al.* Fear induced complexity loss in the electrocardiogram of flight phobics: a multiscale entropy analysis. Biol. Psychol. 73, 272–279 (2006). 16839658 10.1016/j.biopsycho.2006.05.004

[b35] Du X. J., Dart A. M. & Riemersma R. A. Sex differences in the parasympathetic nerve control of rat heart. Clin. Exp. Pharmacol. Physiol. 21, 485–493 (1994). 7982279 10.1111/j.1440-1681.1994.tb02545.x

[b36] McKinley P. S. *et al.* The impact of menstrual cycle phase on cardiac autonomic regulation. Psychophysiol. 46, 904–911 (2009). 10.1111/j.1469-8986.2009.00811.x PMC4451597 19386049

[b37] Leicht A. S., Hirning D. A. & Allen G. D. Heart rate variability and endogenous sex hormones during the menstrual cycle in young women. Exp. Physiol. 88, 441–446 (2003). 12719769 10.1113/eph8802535

[b38] Sacher J., Neumann J., Okon-Singer H., Gotowiec S. & Villringer A. Sexual dimorphism in the human brain: evidence from neuroimaging. Magnet. Res. Imag. 31, 366–375 (2013). 10.1016/j.mri.2012.06.007 22921939

[b39] Ahmadi K., Ahmadlou M., Rezazade M., Azad-Marzabadi E. & Sajedi F. Brain activity of women is more fractal than men. Neurosci. Lett. 535, 7–11 (2013). 23313595 10.1016/j.neulet.2012.12.043

[b40] Jaušovec N. & Jaušovec K. Resting brain activity: differences between genders. Neuropsychologia 48, 3918–3925 (2010). 20875436 10.1016/j.neuropsychologia.2010.09.020

[b41] Yang A. C. *et al.* Complexity of spontaneous BOLD activity in default mode network is correlated with cognitive function in normal male elderly: a multiscale entropy analysis. Neurobiol. Aging 34, 428–438 (2013). 22683008 10.1016/j.neurobiolaging.2012.05.004

[b42] Gillies G. E. & McArthur S. Estrogen actions in the brain and the basis for differential action in men and women: a case for sex-specific medicines. Pharmacol. Rev. 62, 155–198 (2010). 20392807 10.1124/pr.109.002071 PMC2879914

[b43] Bornas X. *et al.* Complexity of everyday life heart rate dynamics and attentional control in healthy students. Nonlinear dynamics Psychol. Life Sci. 17, 345–360 (2013). 23735491

[b44] Holsen L. M. *et al.* Brain hypoactivation, autonomic nervous system dysregulation, and gonadal hormones in depression: a preliminary study. Neurosci. Let. 514, 57–61 (2012). 22395084 10.1016/j.neulet.2012.02.056 PMC3319257

[b45] Yao Y. *et al.* The increase of the functional entropy of the human brain with age. Sci. Rep. 3, 2553 (2013). 24103922 10.1038/srep02853 PMC3793229

[b46] Lin P. F. *et al.* Correlations between the signal complexity of cerebral and cardiac electrical activity: multiscale entropy analysis. PloS one 9, e87798 (2014). 24498375 10.1371/journal.pone.0087798 PMC3912068

[b47] Hansen A. L., Murison R., Eid J. & Thayer J. F. Heart rate variability and cortisol responses during attentional and working memory tasks in naval cadets. Int. Mar. Health 63, 181–187 (2012). 24595973

[b48] Kudielka B. M. & Kirschbaum C. Sex differences in HPA axis responses to stress: a review. Biol. Psychol. 69, 113–132 (2005). 15740829 10.1016/j.biopsycho.2004.11.009

[b49] Perkiomaki J. S., Zareba W., Badilini F. & Moss A. J. Influence of atropine on fractal and complexity measures of heart rate variability. Ann. Noninvasive Electrocard. 7, 326–331 (2002). 10.1111/j.1542-474X.2002.tb00181.x PMC7027705 12431310

[b50] Lipsitz L. A. Physiological complexity, aging, and the path to frailty. Sci. Aging Know. Environ. 16, pe16 (2004). 10.1126/sageke.2004.16.pe16 15103055

[b51] Zarjam P., Epps J., Lovell N. H. & Chen F. Characterization of memory load in an arithmetic task using non-linear analysis of EEG signals. Conf. Proc. IEEE Eng. Med. Biol. Soc. 3519–3522, doi: 10.1109/EMBC.2012.6346725 (2012). 23366686

[b52] Sokunbi M. O. *et al.* Inter-individual differences in fMRI entropy measurements in old age. Biomed. Eng. IEEE Trans. 58, 3206–3214 (2011). 10.1109/TBME.2011.2164793 21859598

[b53] Liu C. Y. *et al.* Complexity and synchronicity of resting state blood oxygenation level‐dependent (BOLD) functional MRI in normal aging and cognitive decline. J. Mag. Res. Imaging 38, 36–45 (2013). 10.1002/jmri.23961 PMC3610850 23225622

[b54] Raja Beharelle A., Kovačević N., McIntosh A. R. & Levine B. Brain signal variability relates to stability of behavior after recovery from diffuse brain injury. Neuroimage 60, 1528–1537 (2012). 22261371 10.1016/j.neuroimage.2012.01.037 PMC3303989

[b55] Ho Y. L., Lin C., Lin Y. H. & Lo M. T. The prognostic value of non-linear analysis of heart rate variability in patients with congestive heart failure—a pilot study of multiscale entropy. PLoS One 6, 4: e18699 (2011). 10.1371/journal.pone.0018699 PMC3076441 21533258

[b56] Aysin B. & Aysin E. Effect of respiration in heart rate variability (HRV) analysis. Conf Proc. IEEE Eng. Med. Biol. Soc. 1, 1776–9 (2006). 17946068 10.1109/IEMBS.2006.260773

[b57] Kanters J. K., Hojgaard M. V., Agner. E. & Holstein-Rathlou N. H. Influence of forced respiration on nonlinear dynamics in heart rate variability. Am. J Physiol. Reg. 1 272, 49–54 (1997). 10.1152/ajpregu.1997.272.4.R1149 9140014

[b58] Penttila. J. *et al.* Time domain, geometrical and frequency domain analysis of cardiac vagal outflow: effects of various respiratory patterns. Clin. Physiol. 21, 365–76 (2001). 11380537 10.1046/j.1365-2281.2001.00337.x

[b59] Lund. V. *et al.* Instantaneous beat-to-beat variability reflects vagal tone during hyperbaric hyperoxia. Undersea. Hyperb. Med. 30, 29–36 (2003). 12841606

[b60] Radhakrishna R. K., Dutt D. N. & Yeragani. V. K. Nonlinear measures of heart rate time series: influence of posture and controlled breathing. Auton. Neurosci. 83, 148–58 (2000). 11593766 10.1016/s1566-0702(00)00173-9

[b61] Nunan D. *et al.* Validity and reliability of short-term heart-rate variability from the Polar S810. Med. Sci. Sports Exer. 41, 243–250 (2009). 10.1249/MSS.0b013e318184a4b1 19092682

[b62] Eriksen B. A. & Eriksen C. W. Effects of noise letters upon identification of a target letter in a non- search task. Percept. Psychophys. 16, 143–149 (1974).

[b63] Jensen A. R. Individual differences in the Hick paradigm. In Speed of Information-Processing and Intelligence (eds Vernon P. A. ) (Ablex, New Jersey 1987).

[b64] Lorr M. & McNair D. M. Profile of Mood States, Bipolar Form. Educational and Industrial Testing Service (San Diego Calif 1984).

[b65] Cohen S., Kamarck T. & Mermelstein R. A global measure of perceived stress. J. Health. Soc. Behav. 24, 386–396 (1983). 6668417

