# Supplementary material for: We should be using nonlinear indices when relating heart-rate dynamics to cognition and mood
Source: Sci Rep. 2015 Nov 13;5:16619. doi: 10.1038/srep16619 (PMC4643265; doi:10.1038/srep16619)
Supplement: Supplementary Information [file srep16619-s1.pdf]

We should be using nonlinear indices when relating heart-rate dynamics to cognition and mood.

SUPPLEMENTARY INFORMATION

Hayley Young and David Benton

S1A. Calculation of sample entropy

First, a set of length m vectors uj is formed

$$u_j = (\mathrm{RR}_j, \mathrm{RR}_{j+1}, \ldots, \mathrm{RR}_{j+m-1}), \quad j = 1, 2, \ldots N - m + 1 \tag{1}$$

where *m* is the embedding dimension and *N* is the number of measured RR intervals. The distance between these vectors is defined as the maximum absolute difference between the corresponding elements:

$$d(u_j, u_k) = \max\left\{ |\mathrm{RR}_{j+n} - \mathrm{RR}_{k+n}| \,\middle|\, n = 0, \ldots, m-1 \right\}. \tag{2}$$

For each u_j the relative number of vectors u_k for which $d(u_j, u_k) \leq r$ is calculated. This index is denoted with $C^m_j(r)$ and can be written as:

$$C_j^m(r) = \frac{\text{nbr of } \left\{ u_k \,\middle|\, d(u_j, u_k) \leq r \right\}}{N - m} \quad \forall\, k \neq j. \tag{3}$$

The values of $C^m_j(r)$ are averaged to yield:

$$C^m(r) = \frac{1}{N - m + 1} \sum_{j=1}^{N-m+1} C_j^m(r) \tag{4}$$

Finally, sample entropy is calculated as follows:

$$\mathrm{SampEn}(m, r, N) = \ln\left(C^m(r) / C^{m+1}(r) \right). \tag{5}$$

S1B. Visual illustration of the calculation of sample entropy.

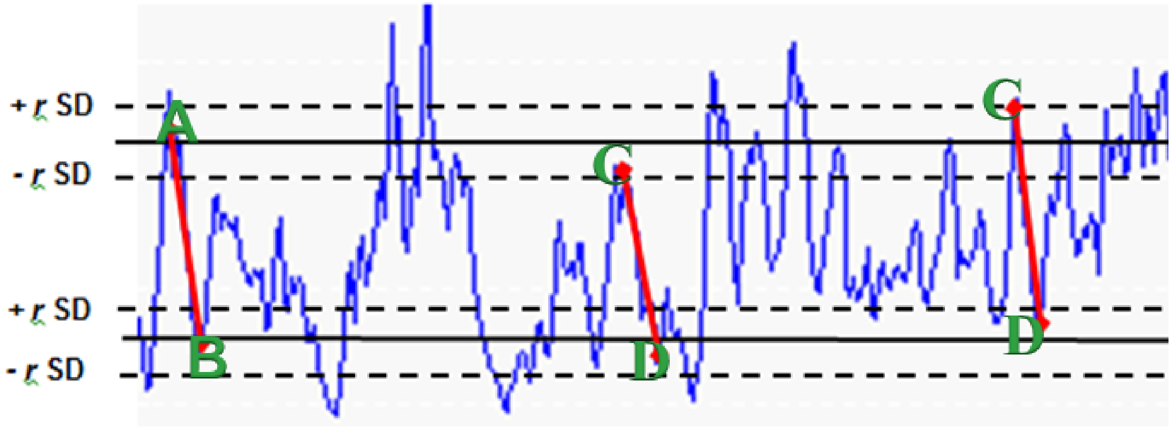

For a two-dimensional vector AB, the tolerance level *r* is represented by dashed lines around point A and B respectively, with width of 2r · SD. Then all vectors, for example CD, whose first and second points (respectively C and D) are within the tolerance ranges of A and B (e.g ±2r · SD), are counted to measure, within a tolerance level r, the regularity of patterns similar to a given pattern of AB. For Approximate Entropy this would be a count of 3 but for Sample Entropy this would be a count of 2. This is because Sample Entropy does not include self-matches and therefore the count is always one less than with Approximate Entropy.

S2A. Detrended fluctuation analysis.

The correlation is extracted for different time scales. First, the RR interval time series is integrated:

$$y(k) = \sum_{j=1}^{k} (\mathrm{RR}_j - \overline{\mathrm{RR}}), \quad k = 1, \ldots, N \tag{6}$$

where RR is the average RR interval. Next, the integrated series is divided into segments of equal length *n*. Within each segment, a least squares line is fitted into the data. Let *yn*(*k*) denote these regression lines. Next the integrated series *y*(*k*) is detrended by subtracting the local trend within each segment and the root-mean-square fluctuation of this integrated and detrended time series is calculated by:

$$F(n) = \sqrt{\frac{1}{N} \sum_{k=1}^{N} (y(k) - y_n(k))^2}. \tag{7}$$

This computation is repeated over different segment lengths to yield the index F(*n*) as a function of segment length *n*. Typically F(*n*) increases with segment length. A linear relationship on a double log graph indicates presence of fractal scaling and the fluctuations can be characterized by scaling exponent α (the slope of the regression line relating log F(*n*) to log *n* **(S2B)**.

S2B. A double log plot of the index F(*n*) as a function of segment length n. α1 and α2 are the short term and long term fluctuation slopes, respectively. Data shown are taken from a female participant in the present study for illustration purposes.

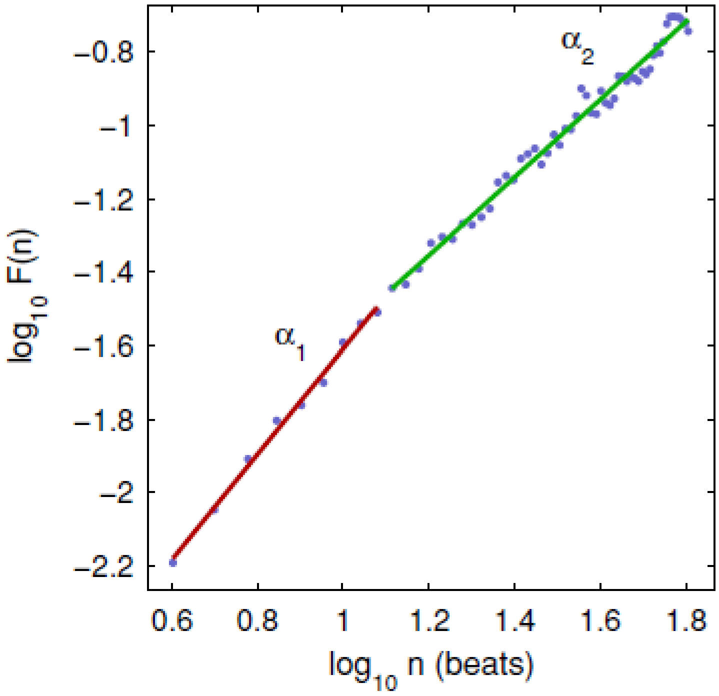

Table S3. False discovery rate procedure.

RANK(j)	P - VALUE	(j/m) x δ	REJECT H_0?
1	0.003	0.003	1
2	0.004	0.004	1
3	0.006	0.006	1
4	0.007	0.008	1
5	0.01	0.01	1
6	0.01	0.01	1
7	0.01	0.01	1
8	0.03	0.01	0
9	0.04	0.01	0
10	0.04	0.02	0
11	0.05	0.02	0
12	0.05	0.02	0
13	0.06	0.02	0
14	0.12	0.02	0
15	0.15	0.03	0
16	0.19	0.03	0
17	0.24	0.03	0
18	0.25	0.03	0
19	0.32	0.03	0
20	0.35	0.04	0
21	0.47	0.04	0
22	0.49	0.04	0
23	0.53	0.04	0
24	0.98	0.05	0

Table S4. Baseline data for males and females. Data are mean (SD). Independent *t* tests found no significant differences between males and females on any of the HR indices.

	MALES	FEMALES
**R-R interval (ms)**	833.0(122.9)	796.3(100.6)
**SDNN (ms)**	69.6(25.4)	69.0(26.4)
**LF power (nu)**	57.7(14.7)	62.7(16.7)
**HF power (nu)**	42.1(14.7)	37.2(16.6)
**LF/HF ratio**	1.8(0.9)	2.2(1.3)
**RPmax (beats)**	208.1(96.6)	251.8(106.8)
**RPrec (%)**	35.7(9.8)	35.6(7.0)
**RPdet (%)**	98.2(1.3)	98.5(0.8)
**RPshen**	3.2(0.3)	3.2(0.2)
**α1**	1.0(0.2)	1.1(0.2)
**SampEn**	1.3(0.4)	1.2(0.2)

R-R interval - The mean of RR intervals, SDNN – Standard deviation of RR intervals, LF – Low frequency, HF – High frequency, RPmax - Maximum line length of diagonal lines in recurrence plot, RPrec - Recurrence rate (percentage of recurrence points in recurrence plot), RPdet - Determinism (percentage of recurrence points which form diagonal lines in recurrence plot), RPshen - Shannon entropy of diagonal line lengths' probability distribution, α1 - Short-term fluctuations of detrended fluctuation analysis, SampEn - Sample entropy.
